# Replication of results from a cervical cancer genome-wide association study in Taiwanese women

**DOI:** 10.1038/s41598-018-33430-x

**Published:** 2018-10-17

**Authors:** Yuh-Cheng Yang, Tzu-Yang Chang, Tze-Chien Chen, Wen-Shan Lin, Chiung-Ling Lin, Yann-Jinn Lee

**Affiliations:** 1 Department of Gynecology and Obstetrics, MacKay Memorial Hospital, Taipei City, Taiwan; 2 Department of Pediatric Endocrinology, MacKay Children’s Hospital, Taipei City, Taiwan; 3 Department of Medical Research, MacKay Memorial Hospital, New Taipei City, Taiwan; 4 Department of Gynecology and Obstetrics, College of Medicine, Taipei Medical University, Taipei City, Taiwan; 5 Department of Pediatrics, School of Medicine, College of Medicine, Taipei Medical University, Taipei City, Taiwan; 6 Institute of Biomedical Sciences, Mackay Medical College, New Taipei City, Taiwan; 7 Department of Medicine, Mackay Medical College, New Taipei City, Taiwan

## Abstract

Genetic epidemiological studies show that genetic factors contribute significantly to cervical cancer carcinogenesis. Several genome-wide association studies (GWAS) have revealed novel genetic variants associated with cervical cancer susceptibility. We aim to replicate 4 GWAS-identified single nucleotide polymorphisms (SNPs), which were associated with invasive cervical cancer in Chinese women, in a Taiwanese population. The *rs13117307 C/T*, *rs8067378 A/G*, *rs4282438 G/T*, and *rs9277952 A/G* SNPs were genotyped in 507 women with cervical squamous cell carcinoma (CSCC) and 432 age/sex matched healthy controls by using TaqMan PCR Assay. Human papillomavirus (HPV) DNA test and typing were performed in CSCC patients. Only the *rs4282438* SNP was found to be significantly associated (*G* allele, odds ratio [OR] = 0.67, *P* = 1.5 × 10^−5^). This protective association remained in HPV-16 positive CSCC subgroup (*G* allele, OR = 0.60, *P* = 1.2 × 10^−5^). In conclusion, our study confirms the association of *rs4282438* SNP with CSCC in a Taiwanese population. However, larger sample sets of other ethnic groups are required to confirm these findings.

## Introduction

Carcinoma of the cervix is the fourth most frequent cancer in women globally. It is also a serious health issue in Taiwan, with approximately 2700 new cases were reported each year^1^. Epidemiologic evidence suggests that human papillomavirus (HPV) is a necessary cause of cervical cancer^2^. Nevertheless, the majority of infected women do not develop the cancer. This indicates that other factors are involved in the cancer progression. Cervical cancer has a strong heritable component and host genetic factors may play an important role in its pathogenesis^3^.

Genome-wide association studies (GWAS) are a systematic approach to identify genes associated with human diseases. This method examines the genetic variations across the genome in different individuals to find out variations that may influence the risk of developing a certain disease. GWAS have successfully discovered many genetic loci associated with complex diseases. As of 2017, the new GWAS catalog lists over 3,000 studies and 58,000 unique single nucleotide polymorphism (SNP) associations examined in over 1,800 various diseases and traits^4^. Cervical cancer, a complex disorder, has also been investigated by GWAS to search potentially associated SNPs. A total of 3 cervical cancer GWAS have been reported in Swedish, Chinese, and mixed European populations and associated cervical cancer loci are at 6p21.3 (*HLA* Class I and II genes), 4q12 (*EXOC1*), and 17q12 (*GSDMB*)^5–7^. Replications of these GWAS results in different ethnic groups are important to substantiate the cervical cancer susceptibility of the genes.

The purpose of this work is to determine whether significant associations identified in the GWAS conducted by Shi *et al*.^6^ can be replicated in a Taiwanese population by using a case-control study with 507 cervical squamous cell carcinoma (CSCC) patients and 432 healthy controls. The reason of choosing the GWAS in the Chinese population for replication is that it included only invasive cervical cancer.

## Results

### HPV distribution of women with CSCC

Among 507 CSCC samples, 370 (73%) had HPV DNA. 242 (65.4%) had HPV 16, 37 (10%) had HPV 18, and the remaining 91 (24.6%) had other types.

### Association of genetic polymorphisms with CSCC

The genotype and allele frequencies of all studied SNPs were successfully determined in 432 controls and 507 CSCC patients (Tables 1–3). No deviation from Hardy-Weinberg equilibrium was observed in the controls except for SNP *rs8067378*. We therefore excluded this SNP from further analysis. We found significant differences in the distribution of genotypes (*P* = 7.1 × 10^−6^) and alleles (*P* = 1.5 × 10^−5^) of SNP *rs4282438 G/T* between controls and CSCC patients (Table 2). The differences remained significant after Bonferroni correction (*P*_*c*_ = 2.1 × 10^−5^ for genotype and *P*_*c*_ = 4.5 × 10^−5^ for allele frequencies). The *G/T* genotype (OR = 0.66, 95% CI 0.51–0.87) and *G* allele (OR = 0.67, 95% CI 0.55–0.80) were significantly less frequent in CSCC patients than in the controls. For the other two SNPs, genotype and allele frequencies did not differ significantly (Tables 1 and 3).

**Table 1:** Genotype and allele frequencies of the *rs13117307 C/T* polymorphism in controls, women with CSCC, and those with HPV-16 positive CSCC^*^.

	Controls (N = 432)	CSCC (N = 507)	HPV-16 positive CSCC (N = 242)	CSCC		HPV-16 positive CSCC	
	n (%)	n (%)	n (%)	*P* value	OR (95% CI)	*P* value	OR (95% CI)
Genotype				0.33		0.46	
C/C	350 (81.0)	395 (77.9)	188 (77.7)		0.83 (0.59–1.15)		0.82 (0.54–1.22)
C/T	77 (17.8)	101 (19.9)	49 (20.2)		1.15 (0.82–1.62)		1.17 (0.77–1.78)
T/T	5 (1.2)	11 (2.2)	5 (2.1)		1.89 (0.60–6.30)		1.80 (0.45–7.24)
Dominant model				0.24		0.30	
Additive model				0.17		0.24	
Allele				0.18		0.27	
T	87 (10.1)	123 (12.1)	59 (12.2)		1.23 (0.91–1.67)		1.24 (0.86–1.79)

^*^CSCC = cervical squamous cell carcinoma; HPV = human papillomavirus; OR = odds ratio; CI = confidence interval.

**Table 2:** Genotype and allele frequencies of the *rs4282438 G/T* polymorphism in controls, women with CSCC, and those with HPV-16 positive CSCC^*^.

	Controls (N = 432)	CSCC (N = 507)	HPV-16 positive CSCC (N = 242)	CSCC		HPV-16 positive CSCC	
	n (%)	n (%)	n (%)	*P* value	OR (95% CI)	*P* value	OR (95% CI)
Genotype				7.1 × 10^−6^		4.1 × 10^−6^	
T/T	109 (25.2)	204 (40.2)	106 (43.8)		2.00 (1.49–2.67)		2.31 (1.63–3.27)
G/T	235 (54.4)	224 (44.2)	102 (42.1)		0.66 (0.51–0.87)		0.61 (0.44–0.85)
G/G	88 (20.4)	79 (15.6)	34 (14.1)		0.72 (0.51–1.02)		0.64 (0.42–0.98)
Dominant model				1.2 × 10^−6^		7.0 × 10^−7^	
Additive model				1.5 × 10^−5^		7.8 × 10^−6^	
Allele				1.5 × 10^−5^		1.2 × 10^−5^	
G	411 (47.6)	382 (37.7)	170 (35.1)		0.67 (0.55–0.80)		0.60 (0.47–0.76)

^*^CSCC = cervical squamous cell carcinoma; HPV = human papillomavirus; OR = odds ratio; CI = confidence interval.

**Table 3:** Genotype and allele frequencies of the *rs9277952 A/G* polymorphism in controls, women with CSCC, and those with HPV-16 positive CSCC^*^.

	Controls (N = 432)	CSCC (N = 507)	HPV-16 positive CSCC (N = 242)	CSCC		HPV-16 positive CSCC	
	n (%)	n (%)	n (%)	*P* value	OR (95% CI)	*P* value	OR (95% CI)
Genotype				0.44		0.23	
G/G	136 (31.5)	178 (35.1)	92 (38.0)		1.18 (0.89–1.56)		1.34 (0.95–1.88)
A/G	209 (48.4)	226 (44.6)	105 (43.4)		0.86 (0.66–1.12)		0.82 (0.59–1.14)
A/A	87 (20.1)	103 (20.3)	45 (18.6)		1.01 (0.73–1.41)		0.91 (0.60–1.38)
Dominant model				0.24		0.09	
Additive model				0.47		0.16	
Allele				0.48		0.17	
A	383 (44.3)	432 (42.6)	195 (40.3)		0.93 (0.77–1.12)		0.85 (0.67–1.07)

^*^CSCC = cervical squamous cell carcinoma; HPV = human papillomavirus; OR = odds ratio; CI = confidence interval.

### Synergy between HPV-16 and genetic polymorphisms on CSCC

Based on the data of HPV-16 positive CSCC, the combined effect of HPV-16 and the genotypes or alleles of the investigated SNPs on CSCC can be investigated. Only SNP *rs4282438 G/T* showed significant difference in the genotype distribution (*P* = 4.1 × 10^−6^) with OR (95% CI) = 0.64 (0.42–0.98) for genotype *G/G*; 0.61 (0.44–0.85) for genotype G/T; 2.31 (1.63–3.27) for genotype *T/T* and in the allele distribution (*P* = 1.2 × 10^−5^) with OR = 0.60 (0.47–0.76) for allele G. Significant differences remained after Bonferroni correction (*P*_*c*_ = 1.2 × 10^−5^ and 3.6 × 10^−5^ for the genotype and allele frequencies, respectively) (Table 2).

## Discussion

In this study, we aimed to replicate the associations between cervical cancer and genetic polymorphisms found in the GWAS on Han Chinese in a Taiwanese population. We found that genotype *G/T* and allele *G* of SNP *rs4282438* were associated with decreased risk of CSCC. These confirm the Chinese GWAS results^6^, in which the *rs4282438 G* allele was reported to be protective against cervical cancer. In addition, we performed the subgroup analysis in HPV-16 positive CSCC women. The results showed that genotype *rs4282438 G/T* and allele *G* still conferred protective effects. The associations of *rs13117307 T* and *rs9277952 A* alleles, although showed the similar trends as those identified in the Chinese GWAS, failed to be replicated in our study. Confirmation of the association between SNP *rs4282438* and CSCC further emphasizes its important role in cervical cancer tumorigenesis.

GWAS has opened a new avenue for studying cervical cancer in an unbiased and hypothesis-free way, which could lead to the previously unsuspected discoveries of susceptibility or resistance genes. The first GWAS of cervical cancer on mostly Swedish patients with cervical intraepithelial neoplasia (CIN) III revealed that *rs2516448 C/T* (near *MICA*), *rs9272143 T/C* (between *HLA-DRB1* and *DQA1*), and *rs3117027C/A* (at *HLA-DPB2*) are associated with CIN III^5^. They also confirmed previously reported associations with HLA-B*07:02, DRB1*15:01-DQB1*06:02, and DRB1*13:01-DQA1*01:03-DQB1*06:03^8–14^. A Chinese GWAS reported invasive cervical cancer to be associated with *rs13117307 C/T* (*EXOC1*), *rs8067378 A/G* (*GSDMB*), *rs4282438 G/T* (between *HLA-DPB1* and *DPB2*), and *rs9277952 A/G* (intergenic)^6^. The most recent GWAS performed by Leo *et al*. demonstrated that HLA-DRB1*15:01-DQB1*06:02-DQA1*01:02, DRB1*04:01-DQA1*03:01, and DRB1*13:01-DQB1*06:03 haplotypes are associated with cervical cancer (CIN II, III, and invasive cancer) in a mixed European population^7^.

SNP *rs4282438*, located in the *HLA-DPB1/2* region, has been proven to be a cervical cancer association variant in both Chinese and Taiwanese populations. Successful replication of the association between *rs4282438* and CSCC in the Taiwanese population not only provides convincing statistical evidence but also extend the generalizability for the association. The class II HLA-DP molecules are heterodimers of alpha and beta chains. They are on antigen-presenting cells and play a central role in the immune system by presenting antigens to CD4^+^ T cells. The antigen binding specificities of the HLA-DP molecules are determined by the highly polymorphic exon 2 region. Genetic associations have been observed with *HLA-DPB1* gene in cervical cancer in Chinese and Taiwanese populations using case-control study^15–17^. The HLA-DPB1*02:02 and *13:01 alleles confer a risk of cervical cancer but DPB1*05:01 allele renders protection against the disease. In addition, analyses of the linkage disequilibrium (LD) between *rs4282438 G/T* and *HLA-DPB1* alleles in the Taiwanese population^17,18^ have shown that *rs4282438 G* allele is highly correlated with DPB1*05:01 allele (D’ = 0.94, r^2^ = 0.83 in controls). This finding may imply that association of *rs4282438 G* allele with cervical cancer is due to the LD with DPB1*05:01.

It is not uncommon to find out discrepancies between GWAS and follow-up replication studies. Although we and Jia *et al*.^19^ showed the association between *rs4282438* and cervical cancer is in agreement with the Chinese GWAS, this association has not been replicated in Swedish^20^ and Japanese populations^21^. The associations of *rs13117307* and *rs8067378* with invasive cervical cancer in the Chinese women was not validated in our study, but have been replicated in Japanese^21^ and Polish populations^22,23^. Additionally, the association of *rs9277952* observed in the Chinese GWAS was unable to be replicated in our study and others^20,21^. A number of potential reasons could explain the inconsistency. These include insufficient statistical power in the replication studies, different minor allele frequencies, LD structures, cancer types and stages, and ethnic backgrounds between the studies^24–26^. Therefore, these confounding factors should be taken into consideration to objectively interpret the replication results.

In conclusion, this is the first replication study of Chinese GWAS on invasive cervical cancer in a Taiwanese population. Our study confirmed the association of *rs4282438* SNP and found this variant was still associated with the HPV-16 positive CSCC women. However, further investigations with large-scale cohorts of different ethnicities are needed to further validate the associations of these SNPs with cervical cancer.

## Methods

### Study subjects

The patients were 507 unrelated women with CSCC (mean age at diagnosis 50.2 ± 10.4 years). CSCC was histologically confirmed on biopsies or resected specimens of cervix. Four hundred and thirty-two age-matched controls (mean age at sampling 51.7 ± 11.5 years) were women with normal Pap smear and no history of cervical dysplasia. All patients and controls had not been included in any previous cervical cancer GWAS. The Institutional Review Board of Mackay Memorial Hospital approved the study and all subjects gave written informed consent. The study was conducted in accordance with the principles expressed in the Declaration of Helsinki.

### DNA extraction

Genomic DNA was extracted from formalin-fixed, paraffin-embedded tissue blocks of CSCC patients and from cervical scrapings of controls using the Qiagen Kit (Qiagen, Valencia, CA).

### HPV Detection and Typing

Polymerase chain reaction (PCR) was applied for HPV DNA detection on CSCC patients. The GP6 + /MY11 PCR primer pair was adopted to generate an approximately 192 bp fragment within the L1 region of HPV genome^27,28^. HPV genotype was then defined by sequencing the PCR product on ABI 377 (Applied Biosystems, Foster City, CA). HPV testing did not perform for the controls.

### SNP genotyping

Given that our study group included only CSCC patients, we selected for replication those SNPs with a genome-wide significant association in the Chinese GWAS^6^. A total of 4 polymorphisms were chosen for genotyping: *rs13117307 C/T* in 4q12 (*EXOC1*), *rs8067378 A/G* in 17q12 (near *GSDMB*), *rs4282438 G/T* in 6p21.32 (between *HLA-DPB1* and *DPB2*), *rs9277952 A/G* in 6p21.32 (intergenic). Genotyping of the replicated SNPs were performed using TaqMan Allelic Discrimination Assay (Applied Biosystems, Foster City, CA) as previously described^29^.

### Statistical analysis

The χ^2^ test was used to evaluate Hardy-Weinberg equilibrium for genotypes in both control and study groups and to assess differences in genotype/allele distributions between the 2 groups. Odds ratios (OR) and 95% confidence intervals (CI) were also determined. The dominant genetic model compared a combination of variant allele homozygote and heterozygote to the wild-type homozygote. The additive genetic model was performed by using Cochran-Armitage trend test function in R package. Bonferroni correction was adopted to calculate corrected *P* (*P*_*c*_) values. Two-tailed *P*_*c*_ values < 0.05 were considered statistically significant.

Using the Quanto Ver. 1.1 software (Department of Preventive Medicine, University of Southern California, CA, USA), we designed the study to have >95% power at a 5% significance level to determine a genotype relative risk of 1.5 of each SNP with an estimated CSCC prevalence of 360/100,000^30^.
